# Cerebellar Transcranial Direct Current Stimulation in People with Parkinson’s Disease: A Pilot Study

**DOI:** 10.3390/brainsci10020096

**Published:** 2020-02-11

**Authors:** Craig D. Workman, Alexandra C. Fietsam, Ergun Y. Uc, Thorsten Rudroff

**Affiliations:** 1 Department of Health and Human Physiology, University of Iowa, Iowa City, IA 52242, USA; alexandra-fietsam@uiowa.edu (A.C.F.); thorsten-rudroff@uiowa.edu (T.R.); 2 Department of Neurology, University of Iowa Hospitals and Clinics, Iowa City, IA 52242, USA; ergun-uc@uiowa.edu

**Keywords:** tDCS, balance, gait, intensity

## Abstract

People with Parkinson’s disease (PwPD) often experience gait and balance problems that substantially impact their quality of life. Pharmacological, surgical, and rehabilitative treatments have limited effectiveness and many PwPD continue to experience gait and balance impairment. Transcranial direct current stimulation (tDCS) may represent a viable therapeutic adjunct. The effects of lower intensity tDCS (2 mA) over frontal brain areas, in unilateral and bilateral montages, has previously been explored; however, the effects of lower and higher intensity cerebellar tDCS (2 mA and 4 mA, respectively) on gait and balance has not been investigated. Seven PwPD underwent five cerebellar tDCS conditions (sham, unilateral 2 mA, bilateral 2 mA, unilateral 4 mA, and bilateral 4 mA) for 20 min. After a 10 min rest, gait and balance were tested. The results indicated that the bilateral 4 mA cerebellar tDCS condition had a significantly higher Berg Balance Scale score compared to sham. This study provides preliminary evidence that a single session of tDCS over the cerebellum, using a bilateral configuration at a higher intensity (4 mA), significantly improved balance performance. This intensity and cerebellar configuration warrants future investigation in larger samples and over repeated sessions.

## 1. Introduction

Parkinson’s disease (PD) is the second most common neurodegenerative disorder and affects approximately 1 million people in the US [1], with total annual health care costs approaching 11 billion dollars [2]. Gait and balance problems affect the majority of people with PD (PwPD) [3] and substantially impact their independence and quality of life [4]. Current pharmacological and surgical PD treatments are either only mildly effective, expensive, or associated with a variety of side-effects [5]. Additionally, rehabilitative and physical activity interventions for gait and balance in PD have only shown mild to moderate positive effects [6,7].

Transcranial direct current stimulation (tDCS) is a non-invasive brain stimulation technique that can modulate cortical excitability [8] and facilitate neural plasticity to improve motor function [9]. Unfortunately, the effects of tDCS in PD are ambiguous [10,11,12,13,14,15,16,17,18] and this disparity might stem from the different tDCS protocols employed (stimulation site, electrode placement/polarity, and stimulation duration, timing, and intensity). For example, stimulated brain areas were the primary motor cortex (M1) [12,13,14,15,16], the dorsolateral prefrontal cortex (DLPFC) [10,11,13,18], and the supplementary motor area (SMA) [17]. However, the cerebellum, which is also dysfunctional in PD [19,20], may represent a viable tDCS brain target for PwPD. The cerebellum plays an important role in coordinating and executing movement (e.g., gait and balance) and is connected to a variety of cortical and basal ganglia areas [19,21]. It is easily stimulated with tDCS [22,23] and has been called a potential “window” for affecting a range of neural functions [23]. Nevertheless, none have investigated the effects of cerebellar tDCS (ctDCS) on gait and balance in PwPD.

Another potential methodological confound between tDCS studies is electrode configuration. Some have used ‘traditional’ unilateral electrode configurations (e.g., anode over M1, cathode over the contralateral supraorbital area), while others have applied bilateral montages (e.g., anode over more-affected M1, cathode over less-affected M1) [9]. Although there is evidence that bilateral montages may be superior to unilateral montages [24,25,26], direct comparisons of these configurations are limited. In addition, despite many tDCS devices having the capability of a range of stimulation intensities, the applied intensities in most tDCS research are ≤ 2 mA [27]. Thus, the effects of higher intensity (4 mA) tDCS on motor function has received little attention [28,29]. Nevertheless, the tolerability of 4 mA tDCS has been shown to be comparable to sham [30,31] or 2 mA [29] and a recent review concluded that there was no evidence that tDCS up to 4 mA for 30 min had any serious negative side effects [27]. Studies exploring the differences in performance between lower (2 mA) and higher (4 mA) intensities are necessary to determine if increasing intensity enhances the desired outcome. Furthermore, repeated sessions of tDCS might have additive effects and further enhance performance [32]. However, preliminary investigations of single applications are important for informing multiple session studies.

Therefore, the purpose of this preliminary investigation was to determine the effects of a single application of combinations of two ctDCS montages (unilateral vs bilateral) and two stimulation intensities (2 mA vs 4 mA) on gait and balance in PD. It was hypothesized that all stimulation montages would improve gait and balance, compared to sham. Furthermore, it was hypothesized that bilateral 4 mA ctDCS would have the greatest improvements compared with the other ctDCS configurations (sham, unilateral 2 mA, bilateral 2 mA, unilateral 4 mA).

## 2. Materials and Methods

Seven PwPD were recruited for this pilot investigation (Table 1). Subjects were recruited from the community and had a positive diagnosis of PD from a movement disorder specialist. A double blind, randomized, sham-controlled, crossover design was employed. This study was approved by the University of Iowa’s Institutional Review Board and all subjects provided written informed consent prior to participation. (Registered on clinicaltrials.gov as NCT04046055.)

Each subject completed five randomly-ordered ctDCS sessions (sham, unilateral 2 mA, unilateral 4 mA, bilateral 2 mA, and bilateral 4 mA) separated by at least 5 days, which provided ample time for excitability changes from tDCS to dissipate [33,34]. Sessions were comprised of gait and balance tests organized into task blocks. The gait block included the 25 ft walk test (25FWT) [35,36], the timed up and go (TUG) test [37,38], and the 6-min walk test (6MWT) [38,39]. The balance block included the Berg Balance Scale (BBS) [38,40] and static posturography [41]. Posturography was performed on a force platform (BTrackS, Balance Tracking Systems, Inc., San Diego, CA) for 60 s with the eyes open and the arms folded, in firm (directly on the force plate) and foam (6 cm Airex Balance Pad Elite (Airex AG, Sins, Switzerland)) conditions. The order of the task blocks was randomized and the tasks within each block were also randomized for each visit. An online randomizer (www.randomizer.org) was used for all randomizations (tDCS conditions, task blocks, and tasks within a block).

**Figure 1:**
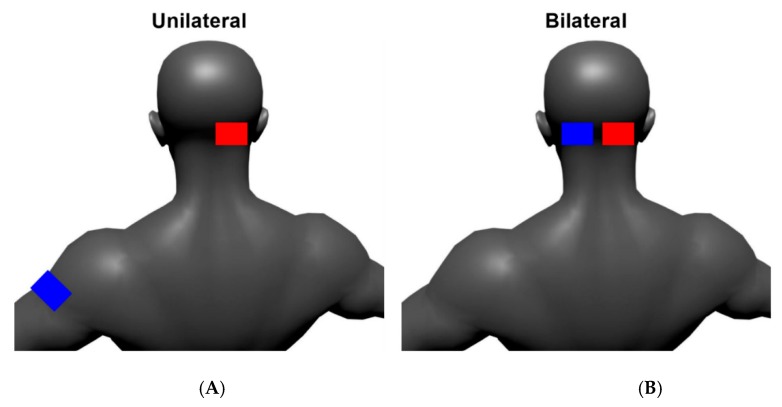
Anode (red) and cathode (blue) electrode configurations for the unilateral (**A**) and bilateral (**B**) montages.

A battery-powered device (Soterix Medical Inc., New York, NY, USA) delivered tDCS via two carbon electrodes placed inside saline soaked sponges (5 cm × 7 cm: 35 cm^2^ surface area). The current densities were 0.06 mA/cm^2^ (2 mA) and 0.11 mA/cm^2^ (4 mA). The 2 mA intensity has been extensively investigated [27], but intensities ≥ 4 mA may be required for the stimulation to reliably reach the brain [42] and studies of higher intensity tDCS are needed [28]. The medial edge of the anode was always positioned 1 cm below and 2 cm lateral to the inion over the cerebellar hemisphere contralateral to the more PD-affected side [43]. The cathode was either located over the contralateral upper arm (unilateral montage) [44] or with the medial edge 1 cm below and 2 cm lateral to the inion over the cerebellar hemisphere ipsilateral to the more PD-affected side [45] (bilateral montage) (Figure 1). Stimulation was administered for 20 min with the subject seated comfortably in a chair [46]. Active stimulation included a 30 s ramp-up to the target intensity (2 mA or 4 mA), after which the intensity was maintained for 20 min before being ramped-down to 0 mA over 30 s. For sham, the tDCS device automatically administered a 30 s ramp-up immediately followed by a 30 s ramp-down both at the beginning and the end of the 20 min stimulation period; in the intervening time, the intensity was maintained at 0 mA. Additionally, because performing a motor task after stimulation might be better than during stimulation [46], the first testing block began 10 min after [47,48] the stimulation ended.

After stimulation, but before beginning the first testing block, stimulation tolerability was assessed by asking the subjects to describe any sensations experienced at the beginning, middle, and end of the 20 min stimulation window. The severity of each reported sensation was rated on a 10-point Likert-type scale (1 = low, 10 = high) [30]. In addition, blinding integrity was evaluated by asking the subjects to guess which stimulation intensity they experienced. Feedback about guesses was only provided after all testing sessions were completed (i.e., the end of Session 5).

A repeated-measures ANOVA (rmANOVA), with tDCS condition (sham vs unilateral 2 mA vs bilateral 2 mA vs unilateral 4 mA vs bilateral 4 mA) as the within-subject factor, was performed on all of the gait and balance outcome measures. In addition, because approximately 50% of subjects might be classified as ‘tDCS responders’ [49], outcomes that were significant in this initial analysis were used to group subjects into ‘responders’ and ‘non-responders.’ Responders were classified as those subjects that showed a tDCS-related improvement on these significant outcomes. Then, another rmANOVA, with tDCS condition (sham vs unilateral 2 mA vs bilateral 2 mA vs unilateral 4 mA vs bilateral 4 mA) as the within-subject factor, was performed on responders and non-responders for that outcome. Paired *t*-tests and effect sizes (Cohen’s **d**) clarified any significant pairwise differences. The normality and sphericity assumptions for the rmANOVA were investigated with the Shapiro-Wilk test and Mauchly’s Test of Sphericity, respectively. Greenhouse-Geisser corrections were used when the sphericity assumption was violated. Initial significance was accepted at *p* ≤ 0.05 and adjusted with Bonferroni corrections for the *post hoc* tests. Analysis was performed in SPSS 25 (IBM Corp, Armonk, NY, USA).

## 3. Results

**Figure 2:**
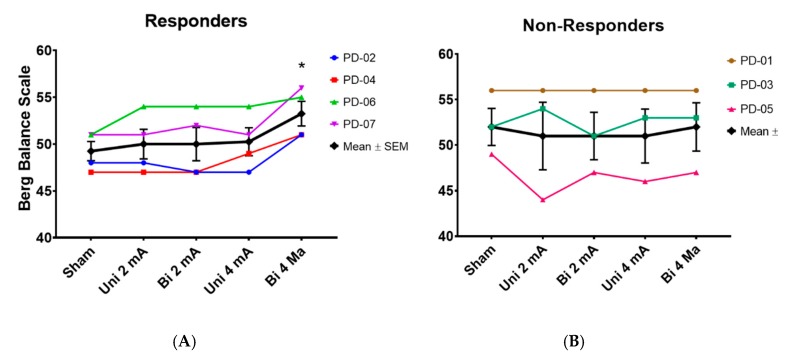
Berg Balance Scale scores in different transcranial direct current stimulation conditions for subjects classified as ‘responders’ (**A**) and ‘non-responders’ (**B**). * = Bi 4 mA significantly different from sham. Uni = unilateral montage; Bi = bilateral montage.

All of the subjects completed all of the testing conditions and no data were missing or removed. None of the subjects used assistive devices. All of the assumptions for the rmANOVA were met and no corrections were made. The results of the first rmANOVA revealed that only the BBS was statistically significant (F(4,24) = 3.222, *p* = 0.030). After grouping the subjects into responders and non-responders, the follow-up rmANOVA was still significant for the responders (F(4,12) = 9.0, *p* = 0.001), but not for the non-responders (F(4,8) = 0.281, *p* = 0.66). Pairwise testing of the responders revealed that BBS scores in the bilateral 4 mA condition were significantly higher than sham (*p* = 0.002, **d** = 1.69) (Figure 2).

The sensations most commonly reported in any tDCS configuration were burning, itching, tingling, and pins/needles. The severity of these sensations was generally mild (i.e., ≤ 3.5), although there were some subjects (*n* = 2) that reported moderate sensations (i.e., ≤ 5) at the beginning of both 4 mA conditions (unilateral and bilateral). Importantly, all sensations, including the most moderate, reduced or disappeared from the beginning to the end of the stimulation (e.g., from 5 to 2). For blinding integrity, none of the subjects correctly guessed the sham condition (guessed sham = 0; guessed 2 mA = 5; guessed 4 mA = 2), a majority correctly guessed 2 mA in the unilateral condition (guessed sham = 3; guessed 2 mA = 4; guessed 4 mA = 0) and the bilateral condition (guessed sham = 2; guessed 2 mA = 4; guessed 4 mA = 1), and a minority correctly guessed 4 mA in the unilateral condition (guessed sham = 1; guessed 2 mA = 5; guessed 4 mA = 1) and bilateral condition (guessed sham = 0; guessed 2 mA = 4; guessed 4 mA = 3). Furthermore, it is noted that two of the subjects guessed 2 mA for every condition, indicating a low confidence in discriminating between conditions. A more comprehensive tolerability report is presented in the Supplementary Table S1.

## 4. Discussion

The purpose of this pilot study was to determine the effects of ctDCS at different intensities (2 mA and 4 mA) using different configurations (unilateral and bilateral). The results indicated a significant improvement in balance performance with the bilateral 4 mA condition compared to sham (~4 points change in BBS; minimal clinically important difference = 5 points [7]), but no significant changes in gait or other balance measures. Because the cerebellum has a strong role in balance control [50], the effect on a clinical balance measure like the BBS, that includes both static and dynamic balance tasks, is not surprising. The lack of significant changes in gait or the static posturography variables may be from the small number of subjects included in this pilot investigation. This study provides preliminary and supporting evidence for several important tDCS topics. First, these results offer initial evidence for the cerebellum as a viable stimulation site to affect balance in PwPD. Although others have previously applied various non-invasive cerebellar stimulation techniques in PD (i.e., repetitive transcranial magnetic stimulation (rTMS), theta burst stimulation (TBS), or tDCS), these investigations were all in the context of levodopa-induced dyskinesias [51]. Second, this study supports the concept that bilateral montages might improve performance more than unilateral montages [26], which could be the result of bilateral montages increasing the activity of both brain hemispheres compared with unilateral montages [24,25]. Third, this investigation suggests that higher intensities might be required to more reliably affect cortical activity. A recent study also indicated the necessity of using higher intensities (4–6 mA) to get sufficient current past the shunting tissues (scalp, subcutaneous tissue, skull) to the brain to influence excitability [42]. Interestingly, in the present study a combination of a bilateral montage and a higher intensity (4 mA) was required in order to elicit a significant balance response in the majority of the subjects. This is particularly noteworthy because many of the responders in this study likely would have been classified as non-responders if either of the ‘standard’ configurations (unilateral) or intensities (2 mA) were used (Figure 2). Fourth, this study adds to the early, but growing, evidence of the tolerability of 4 mA stimulation intensities [29,30,31,52,53,54].

The most prominent limitation of this study is the small number of subjects, which necessitates caution when interpreting the results. In addition, the mechanistic underpinnings of the results, as clarified using TMS [55], electroencephalography (EEG) [55], or neuroimaging [51] (e.g., positron emission tomography [PET], functional magnetic resonance imaging [fMRI]) were not investigated and therefore remain ambiguous. Lastly, this study did not assess the duration of the effects of ctDCS. Previous studies have shown that a single session of lower intensity (2 mA) stimulation might last as long as 90–120 min [34], but the duration of higher intensity stimulation remains uncertain.

Future studies should continue to explore, compare, and contrast ctDCS with other stimulation targets (e.g., M1). Furthermore, mechanistic exploration (e.g., PET imaging) of the differences between higher and lower tDCS intensities, over diverse stimulation sites, and in unilateral and bilateral configurations are also suggested. In addition, because repeated tDCS sessions might induce additive effects [32], are more likely to elicit positive findings [56], and have promising preliminary results [57], future investigations of the short- and long-term effects of multiple sessions of tDCS in neurological populations is of high interest. Lastly, a barrier to all tDCS research is the a priori identification of responders and non-responders. Future investigations exploring the influence of demographic, anatomical, or disease-related variables on responses to tDCS would greatly enhance this growing field.

## 5. Conclusions

In summary, this study provides preliminary evidence that a single session of tDCS over the cerebellum, using a bilateral configuration at a higher intensity (4 mA), significantly improved balance performance compared to sham. This intensity and montage (stimulation site and electrode configuration) warrants future investigation in a larger sample, especially with repeated bouts of stimulation (e.g., daily for five days).

## Figures and Tables

**Table 1:** Subject demographic information. Data are mean ± SD.

Sex (male/female)	5/2
Age (years)	72.4 ± 6.4
Height (cm)	172.4 ± 12.9
Weight (kg)	81.3 ± 21.7
Time since diagnosis (years)	4.3 ± 2.5
Telephone-Montreal Cognitive Assessment	19.3 ± 2.1
MDS-UPDRS Part III	32.6 ± 14.2
Hoehn and Yahr Scale	1.9 ± 0.4
Levodopa Equivalent Daily Dose	889.8 ± 497.7

## Supplementary Materials

The following are available online at https://www.mdpi.com/2076-3425/10/2/96/s1, Table S1: Sensation and blinding results, stratified by stimulation intensity and time window during stimulation (beginning, middle, end).
