# Supplementary material for: Cerebellar Transcranial Direct Current Stimulation in People with Parkinson’s Disease: A Pilot Study
Source: Brain Sci. 2020 Feb 11;10(2):96. doi: 10.3390/brainsci10020096 (PMC7071613; doi:10.3390/brainsci10020096)
Supplement: Supplementary file 1 [file brainsci-10-00096-s001.pdf]

Table S1. Sensation and blinding results, stratified by stimulation intensity and time window during stimulation (beginning, middle, end). The range of sensation reports was 1 = ''barely perceptible'' to 10 = "worst I could possibly stand." Data are mean ± SD.

Sensation	**Sham**			**Unilateral 2 mA**			**Bilateral 2 mA**		
	Beginning	Middle	End	Beginning	Middle	End	Beginning	Middle	End
Burn	1.0 ± 0.0 (n = 1)	1.0 ± 0.0 (n = 1)	2.0 ± 1.0 (n = 3)	1.0 ± 0.0 (n = 1)	n/a	n/a	2.5 ± 2.1 (n = 2)	2.0 ± 0.0 (n =1)	n/a
Itch	1.5 ± 0.71 (n = 2)	2.0 ± 0.0 (n = 1)	3.0 ± 0.0 (n = 1)	2.0 ± 0.0 (n = 1)	2.0 ± 0.0 (n = 1)	2.0 ± 0.0 (n = 1)	2.0 ± 0.0 (n =1)	2.0 ± 0.0 (n =1)	2.0 ± 0.0 (n =1)
Tingle	1.0 ± 0.0 (n = 1)	n/a	2.0 ± 0.0 (n = 1)	1.75 ± 0.5 (n = 4)	1.25 ± 0.5 (n = 4)	1.25 ± 0.5 (n = 4)	2.0 ± 0.0 (n =1)	1.0 ± 0.0 (n = 1)	2.5 ± 2.1 (n = 2)
Needles	2.5 ± 0.71 (n = 2)	2.0 ± 0.0 (n = 1)	3.0 ± 0.0 (n = 1)	3.5 ± 2.1 (n = 2)	2.0 ± 0.0 (n = 1)	2.0 ± 0.0 (n = 1)	3.0 ± 0.0 (n = 1)	n/a	n/a
Poking	n/a	n/a	4.0 ± 0.0 (n = 1)	n/a	n/a	n/a	6.0 ± 0.0 (n = 1)	n/a	n/a
Tickle	n/a	n/a	n/a	n/a	2.0 ± 1.4 (n = 2)	1.0 ± 0.0 (n = 1)	n/a	n/a	n/a
Prickle	n/a	n/a	n/a	n/a	n/a	1.0 ± 0.0 (n = 1)	4.0 ± 0.0 (n = 1)	4.0 ± 0.0 (n = 1)	1.0 ± 0.0 (n = 1)
Blinding (n)	sham = 0	2 mA = 5	4 mA = 2	sham = 3	2 mA = 4	4 mA = 0	sham = 2	2 mA = 4	4 mA = 1
	**Unilateral 4 mA**			**Bilateral 4 mA**					
	Beginning	Middle	End	Beginning	Middle	End			
Burn	2.0 ± 1.4 (n = 2)	1.5 ± 0.7 (n = 2)	1.0 ± 0.0 (n = 1)	3.0 ± 1.2 (n = 4)	2.25 ± 1.3 (n = 4)	2.0 ± 1.0 (n = 3)			
Itch	n/a	n/a	n/a	2.0 ± 0.0 (n = 1)	1.0 ± 0.0 (n = 1)	1.0 ± 0.0 (n = 1)			
Tingle	1.5 ± 0.7 (n = 2)	1.5 ± 0.7 (n = 2)	1.0 ± 0.0 (n = 1)	3.0 ± 0.0 (n = 1)	2.0 ± 0.0 (n = 1)	1.0 ± 0.0 (n = 1)			
Needles	4.33 ± 2.08 (n = 3)	1.0 ± 0.0 (n = 1)	1.0 ± 0.0 (n = 1)	5.0 ± 1.4 (n = 2)	3.5 ± 0.7 (n = 2)	2. ± 0.0 (n = 1)			
Poking	7.0 ± 0.0 (n =1)	3.0 ± 0.0 (n = 1)	n/a	n/a	n/a	n/a			
Tickle	n/a	2.0 ± 0.0 (n = 1)	n/a	n/a	n/a	1.0 ± 0.0 (n = 1)			
Prickle	n/a	1.0 ± 0.0 (n = 1)	1.0 ± 0.0 (n = 1)	n/a	n/a	n/a			
Blinding (n)	sham = 1	2 mA = 5	4 mA = 1	sham = 0	2 mA = 4	4 mA = 3			
